# The interplay of aging, genetics and environmental factors in the pathogenesis of Parkinson’s disease

**DOI:** 10.1186/s40035-019-0165-9

**Published:** 2019-08-16

**Authors:** Shirley Yin-Yu Pang, Philip Wing-Lok Ho, Hui-Fang Liu, Chi-Ting Leung, Lingfei Li, Eunice Eun Seo Chang, David Boyer Ramsden, Shu-Leong Ho

**Affiliations:** 1 Division of Neurology, Department of Medicine, Queen Mary Hospital, University of Hong Kong, Hong Kong, People’s Republic of China; 2 Institute of Metabolism and Systems Research, University of Birmingham, Birmingham, UK

**Keywords:** Parkinson’s disease, Genetics, Aging, Environmental toxins

## Abstract

**Background:**

Parkinson’s disease (PD) is characterized by dopaminergic neuronal loss in the substantia nigra pars compacta and intracellular inclusions called Lewy bodies (LB). During the course of disease, misfolded α-synuclein, the major constituent of LB, spreads to different regions of the brain in a prion-like fashion, giving rise to successive non-motor and motor symptoms. Etiology is likely multifactorial, and involves interplay among aging, genetic susceptibility and environmental factors.

**Main body:**

The prevalence of PD rises exponentially with age, and aging is associated with impairment of cellular pathways which increases susceptibility of dopaminergic neurons to cell death. However, the majority of those over the age of 80 do not have PD, thus other factors in addition to aging are needed to cause disease. Discovery of neurotoxins which can result in parkinsonism led to efforts in identifying environmental factors which may influence PD risk. Nevertheless, the causality of most environmental factors is not conclusively established, and alternative explanations such as reverse causality and recall bias cannot be excluded. The lack of geographic clusters and conjugal cases also go against environmental toxins as a major cause of PD. Rare mutations as well as common variants in genes such as *SNCA, LRRK2* and *GBA* are associated with risk of PD, but Mendelian causes collectively only account for 5% of PD and common polymorphisms are associated with small increase in PD risk. Heritability of PD has been estimated to be around 30%. Thus, aging, genetics and environmental factors each alone is rarely sufficient to cause PD for most patients.

**Conclusion:**

PD is a multifactorial disorder involving interplay of aging, genetics and environmental factors. This has implications on the development of appropriate animal models of PD which take all these factors into account. Common converging pathways likely include mitochondrial dysfunction, impaired autophagy, oxidative stress and neuroinflammation, which are associated with the accumulation and spread of misfolded α-synuclein and neurodegeneration. Understanding the mechanisms involved in the initiation and progression of PD may lead to potential therapeutic targets to prevent PD or modify its course.

## Background

Parkinson’s disease (PD) is a neurodegenerative condition characterized clinically by the cardinal motor symptoms of bradykinesia, rest tremor, rigidity and postural instability, and non-motor symptoms such as olfactory dysfunction, constipation, depression and REM sleep behavior disorder (RBD). Pathologically, it is defined by dopaminergic neuronal loss in the substantia nigra pars compacta (SN), and intracellular inclusions called Lewy bodies (LB) in the neurons of affected brain regions. LB are largely composed of misfolded α-synuclein and also include ubiquitin and small amounts of several other proteins. PD is the second most common neurodegenerative disorder after Alzheimer’s disease, and the most common movement disorder, affecting 1% of those over the age of 60 [1]. Aging is the most important risk factor for PD, with incidence rising sharply at the age of 60’s and then exponentially in subsequent decades of life. About 5–10% of patients have familial PD due to Mendelian inheritance of genetic mutations. In addition, genome-wide association studies (GWAS) have identified common genetic variants which contribute to increased PD susceptibility [2]. Nevertheless, the majority of patients have sporadic, or idiopathic, PD. Various environmental factors have been shown to affect the risk of PD. Risk factors include pesticide exposure and traumatic brain injury, and protective factors include tobacco smoking and physical activity [3]. Individually, aging, genetics and environmental factors only explains a small fraction of PD. Therefore, the etiology of most cases of PD is likely to be complex and involve an interplay among aging, genetic susceptibility and environmental factors.

## Main text

### Aging: a pre-parkinsonian state

The association of aging with PD is well described, and aging is recognized to be a primary risk factor for the development of PD [4, 5]. A meta-analysis including 47 epidemiological studies of PD showed a rising prevalence with age, from 41 per 100,000 in those 40–49 years of age, to 1903 per 100,000 in those older than age 80 [6]. The incidence of PD peaks at 70–79 for both men and women, with a decline noted in those 80+, which could be due to mortality competing with PD incidence [7]. Despite epidemiological evidence that aging is a primary risk factor of PD, the biological correlates of this connection are unclear. The importance of aging in the pathogenesis of PD also has implications for the design of animal and cell models of PD and interpretation of findings from these models.

As aging is strongly associated with the development of PD, as suggested by epidemiological studies, there must be shared biological pathways between the two such that age-related changes lay the path to the dopaminergic neurodegeneration seen in PD [8]. Aging is an evolutionarily conserved natural process that involves dysregulation of several pathways, such as oxidative stress, mitochondrial dysfunction, autophagy and neuroinflammation, many of which are also involved in neurodegenerative conditions [9]. In humans, brain weight is greatest in the early teens and remains relatively stable until the fifth decade [10]. From 50 to 90 years of age, the loss of brain weight is approximately 2–3% per decade. Accordingly, a reduction in SN volume with aging has also been reported in humans and monkeys [11, 12]. It has been estimated that the number of SN neurons declines by 7 to 9.8% per decade in healthy individuals without neurological disorders [13, 14].

Neurodegeneration in PD is characterized by more profound loss of neurons in the ventral SN compared with dorsal SN and relative sparing of adjacent ventral tegmental area.

Evidence from rhesus monkeys showed that in ventral SN there is an age-related decline in staining for tyrosine hydroxylase (TH), a histological marker for DA neuron. These appear to be the neurons most vulnerable to degeneration in PD [15]. Furthermore, there is evidence of impaired intracellular clearance mechanisms in aged nonhuman primate brains such as increased ubiquitin-positive inclusions in neurons and accumulation of cytoplasmic lipofuscin, a byproduct of lysosome activity associated with the removal of defective organelles. The former was nearly exclusively found in the most vulnerable ventral SN neurons, whereas the latter was most prominent in ventral SN, which may suggest more efficient removal of damaged organelles in the relatively degeneration-resistant ventral DA neurons [15]. These results are consistent with the view that aging is associated with a region-specific impairment of intracellular clearance mechanisms most predominantly in ventral SN, resulting in a pre-parkinsonian state with increased vulnerability to degeneration of this population of neurons. In accordance with this, a robust age-related increase in intracellular α-synuclein has been demonstrated specifically in the nigral neurons but not the ventral tegmental area of both rhesus monkeys and normal healthy humans [16]. The increase in intracellular α-synuclein was strongly associated with an age-related decrease in nigral TH, again suggesting that aging represents a subthreshold pre-parkinsonian state [16]. The effect of aging on PD pathogenesis is further supported by results from an experiment which showed that delaying aging in the worm *Caenorhabditis elegans (C. elegans)* by introducing a mutation in the gene encoding insulin-like growth factor 1 (*daf-2)* resulted in a doubling of lifespan together with increased survival of DA neurons and amelioration of DA-dependent behavior deficits [17].

Different hypotheses have been put forward to explain the selective vulnerability of nigral neurons to age-related degeneration, including oxidative stress from auto-oxidation of intracellular dopamine that is not effectively sequestered into synaptic vesicles [18], iron accumulation in the SN which induces neuronal damage [19, 20], and increased microglia activation, a marker of neuroinflammation, in the ventral SN compared to other brain regions in response to the neurotoxin 1-methyl-4-phenyl-1,2,3,6-tetrahydropyridine (MPTP) [21, 22].

Although aging is an important factor in the pathogenesis of PD, very few experimental models incorporate aging as a variable. This may be due to difficulty in obtaining and the costs associated with maintaining aged animals. Using human induced pluripotent stem cells (iPSC) also carries the problem of modelling a late-onset disease with rejuvenated cells, thus removing aging as a factor. To this end, a study has shown that induction of in vitro aging in iPSCs, by increasing the expression of proteins related to cell aging, resulted in the phenotypes of DA neurodegeneration such as dendrite shortening and progressive loss of TH [23]. More recently, the annual killifish *Nothobranchius furzeri,* which (i) has a naturally short lifespan of 3 to 6 months, (ii) exhibits age-dependent degeneration of dopaminergic and noradrenergic neurons, and (iii) develops brain accumulation of inclusion bodies containing α-synuclein, has been proposed as a possible animal model to study mechanisms underlying PD which takes aging into account [24].

**Fig. 1:**
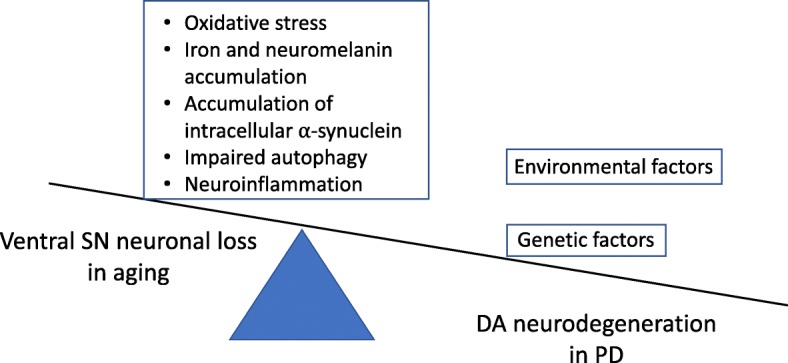
Normal aging is characterized by subclinical SN neuronal decline due to dysregulation in pathways resulting in increased oxidative stress, accumulation of brain iron and neuromelanin, impaired autophagy, accumulation of intracellular α-synuclein and activation of microglia and neuroinflammation. Additional genetic and environmental factors contribute to tip the balance from normal aging to accelerated DA neurodegeneration seen in PD

Although aging is important in the pathogenesis of PD, aging alone is not sufficient to cause PD. The prevalence of PD in those above 80 years of age is around 2% [6], meaning that the majority of people do not develop PD even in old age. Although dopaminergic neuronal degeneration occurs with advancing age, and this may be accompanied with mild parkinsonian signs, the rate of degeneration that occurs in normal aging cannot account for the putaminal dopaminergic loss that is seen in PD. It is thus likely that additional genetic and environmental factors are necessary to tip the balance from subclinical SN neuronal decline in normal aging to accelerated DA neurodegeneration seen in PD (Fig. 1).

### Environmental factors: etiological and disease-modifying effects on PD

From 1817 when Dr. James Parkinson first described 6 patients with the condition that would later bear his name [25], much progress was made in the following 150 years. It became known that the SN was the site affected by PD pathology together with the presence of cytoplasmic inclusions, and levodopa became available as the first effective symptomatic drug treatment of PD in 1960’s [2]. However, the etiology of PD remained elusive.

An important breakthrough took place in 1983 when Langston and colleagues described a group of drug users who developed parkinsonism that was indistinguishable to PD after exposure to MPTP, a contaminant found in synthetic heroin [26]. Although MPTP does not occur in nature, there are other compounds that are chemically similar to MPTP such as paraquat which is found in herbicides. The discovery led to the hypothesis that environmental toxins can cause PD, and efforts were made to identify these factors. Progress in this field has largely been attributed to case-control and prospective longitudinal studies. Indeed, occupational exposure to paraquat has been shown to increase the risk of parkinsonism by almost 3-fold [27]. Similarly, farmers who reported the use of rotenone, an organic pesticide which occurs naturally in seeds and stems of several plants such as *Pachyrhizus erosus* (jicama vine), were 2.5 times more likely to develop PD than non-users [28]. Rotenone is a lipophilic mitochondrial toxin, which acts by inhibiting mitochondrial Complex 1, increasing reactive oxygen species and reducing ATP production. It is used in experimental models of PD [28]. A review which examined 66 meta-analyses involving 691 studies on environmental risk factors for PD reported six environmental factors with highly suggestive association: head injury, anxiety, depression, and beta-blocker usage increase risk of PD, whereas decreased risk is seen with smoking, physical activity and uric acid levels [29]. Another review reported dairy products, pesticides, and traumatic brain injury as risk factors, and smoking, caffeine, urate, physical activity, ibuprofen and calcium channel blockers as protective factors [3] (Table 1).

**Table 1:** Examples of environmental factors and their biologic correlates

Protective factors	Biologic correlates
Smoking	• Nicotine acts at nicotinic acetylcholine receptors to trigger downstream effects that reduce neuronal damage [30]
Physical activity	• Increases serum urate [31] • Increases neurotrophic factors [32]
Urate	• Anti-oxidant by activating of Nrf2/antioxidant response pathway [33]
Ibuprofen	• Anti-inflammatory effect by activation of peroxisome proliferator-activated receptor gamma (PPARγ) [34]
Calcium channel blockers	• Plausible blockage of calcium-channel induced metabolic stress of mitochondria of DA neurons [35]
Caffeine	• Adenosine A_2A_ receptor blockade [36]
Risk factors	
Pesticides	• Mitochondrial toxins, oxidative stress [27, 28]
Dairy	• Urate-lowering effects of dairy products [37]
Traumatic brain injury	• Breakdown of blood-brain barrier, brain inflammation, impaired mitochondrial function, increase in glutamate release, α-synuclein accumulation [38]
Anxiety or depression	• May be prodromal symptom rather than risk factor due to loss of serotonergic neuronal cells in dorsal raphe nucleus in early PD [39]
Beta-blockers	• Aggravate the loss of norepinephrine neurons in locus coeruleus and deficits in norepinephrine in PD [40]

The most robust beneficial environmental factor associated with PD is cigarette smoking which has been seen in early case-control studies and then confirmed in more recent large prospective cohorts. Active smokers have 50% lower risk of PD compared to never smokers [41]. There is a strong dose-response relationship including duration, intensity, pack-years and years since last smoking: PD risk decreases with increasing duration of smoking and increases again with time since quitting [42]. Preliminary reports also support an inverse relationship between passive smoking, smokeless tobacco use and PD [43–45].

Despite the overwhelming epidemiological evidence, the causality between smoking and PD remains controversial. Different alternative hypotheses have been put forward to explain this association. One possibility is that individuals predisposed to PD tend to be risk-aversive with low sensation-seeking score, and thus are less likely to start smoking. However, adjustment for sensation-seeking score only slightly attenuated the inverse relationship between smoking and PD [46]. Reduced responsiveness to nicotine during prodromal phase of PD may make it easier to quit smoking, i.e., reverse causation [47], but this cannot explain why ever-smokers have a lower risk of PD than never-smokers [41]. Mortality due to causes unrelated to PD may also result in an apparent under-representation of smokers in the PD population. Overall, smoking has been found to have a strong and consistent inverse relationship with PD, although confounding factors and reverse causation cannot be totally excluded. The biologic correlate of this protective effect is under investigation. Nicotine has been found to have neuroprotective effects against nigrostriatal damage in animal models of PD possibly by interacting with nicotinic acetylcholine receptors which trigger downstream events to halt neuronal damage [30].

Similar to the difficulty in discerning causality between smoking and PD, the interpretation of how other environmental factors affect the risk of PD remains difficult despite an apparent link between the environment and PD. One major challenge is that PD has a long prodromal period of years if not decades. During this prolonged prodromal stage, many etiological factors may have come into play to affect the risk and progression of PD. There are likely to be recall bias and confounding factors. Furthermore, some environmental factors may change over time as a result of PD development leading to reverse causation: reduced physical capacity, fatigue and pain in prodromal PD may reduce the level of physical activity, resulting in an apparent inverse association between physical activity and PD risk [48]. While mild head injury has not been found to be associated with PD [38], head trauma severe enough to cause loss of consciousness or concussion has been shown to increase the risk of PD in a meta-analysis [49]. Furthermore, higher risk of PD is seen with earlier age of first head injury resulting in loss of consciousness, in accordance to animal models showing that early life brain inflammation is associated with persistent disturbances of the nigrostriatal pathway [50]. However, other studies have shown that the risk of PD is highest soon after traumatic brain injury and then decreases gradually over time, suggesting the possibility of reverse causation: the diagnosis of PD is made during evaluation of head injury which may be due to subtle imbalance in early disease [51, 52]. MPTP is well described to cause parkinsonism which is indistinguishable from PD, and toxins such as paraquat and rotenone clearly cause neuronal damage by disrupting mitochondrial function and increasing oxidative stress. Although these toxins are useful in animal and cell models of PD, most human PD patients have no history of exposure to these compounds and PD had been described well before the height of the industrial revolution when these pesticides were manufactured and used at scale. Thus, they are unlikely to be major etiological factors. In summary, most environmental factors have inconsistent or weak association with PD, with alternative explanations such as reverse causality and recall bias not excluded. The lack of geographic clusters and conjugal cases also go against environmental toxins as a major cause of PD. It is likely that environmental factors alone will not account for the majority of PD.

### Genetics: insights into etiology

Improvement in genetic analysis techniques in the 1990’s led to the discovery of the first genetic cause of PD: mutations in the *SNCA* gene encoding α-synuclein [53]. At around the same time, α-synuclein was found to be the major constituent of LB, the pathological hallmark of PD [54, 55]. Subsequently, multiplications of the *SNCA* gene have been found to cause PD with penetrance increasing with gene dosage [56, 57]. These discoveries brought α-synuclein to center stage in the study of the pathogenesis of PD and led to the hypothesis that during different stages of the disease, α-synuclein spreads in a stereotypical way within the nervous system in a prion-like fashion [58, 59].

Soon after the discovery of *SNCA* mutations as a cause of autosomal dominant PD, genetic studies of familial PD identified additional monogenic causative genes: *LRRK2, VSP35,* and *CHCHD2* causing autosomal dominant PD, and *Parkin, PINK1, DJ-1, ATP13A2, FBXO7* and *PLA2G6* causing autosomal recessive PD [60]. To explore genetic contribution to sporadic disease, a twin study showed that concordance was higher in monozygotic than dizygotic twins in those with disease onset before age 50 but not in those with later onset, suggesting there is a genetic basis at least for young-onset disease [61]. An update of this twin study recently showed heritability of PD to be 27% [62]. In addition, GWAS and genome-wide complex trait analysis (GCTA) uncovered susceptibility loci which predispose to sporadic PD [63, 64]. Of note, variants of some causative genes, e.g. *SNCA* and *LRRK2,* have been found to contribute to risk of PD in sporadic disease, suggesting a role of these genes that is common to both familial and sporadic forms. Furthermore, variants with incomplete penetrance in *GBA* have been shown to be strong risk factors for PD [65]. These findings helped advance the understanding of PD pathogenesis and facilitated development of various genetic animal models to study the role of α-synuclein aggregation and transmission and to identify potential therapeutic strategies to modulate the disease. Of particular interests are the genes *SNCA*, *LRRK2* and *GBA* (Table 2).

**Table 2:** Examples of genes associated with PD risk

Gene	Prevalence in PD	Proposed pathogenic mechanisms	Therapeutic strategies
*SNCA (PARK1)* [53]	• Missense and multiplication mutations are rare and cause monogenic familial PD [59] • Common polymorphisms are risk factors for sporadic PD [64, 66]	• Missense mutations are located in N-terminal region of α-synuclein and cause a variety of structural effects: formation of oligomeric aggregates, loss of membrane binding [67] • Duplications, triplications and common polymorphisms increase α-synuclein expression [56, 57]	• Decrease α-synuclein production and aggregation [68] • Increase α-synuclein degradation by activating autophagy [68] • Decrease extracellular α-synuclein e.g. using α-synuclein antibodies [68] • Inhibit uptake of extracellular α-synuclein [68]
*LRRK2 (PARK8)* [69]	• Present in 40% of familial cases and 10% of sporadic cases [65]	• Monogenic pathogenic mutations either reduce GTPase activity or increases kinase activity [70–72] • Risk variants likely increase LRRK2 activity [70–72]	• LRRK2 kinase inhibitors [73, 74]
*GBA* [75, 76]	• Prevalence varies with ethnicity but is as high as 30% in Ashkenazi Jews and 10% in Chinese and Japanese [77–80]	• Reduction in GCase activity results in accumulation of substrates (e.g. glucosylceramide) and of α-synuclein [81]	• Small molecule chaperones to increase GCase activity [68] • Substrate reduction e.g. glucosylceramide synthase inhibitors [68]

Missense mutations and triplications of *SNCA* cause autosomal PD with high penetrance, whereas duplication mutations have a penetrance of 40% [59]. The discovery that multiplication mutations can cause PD is particularly important as it suggests that over-expression of wild-type α-synuclein is sufficient to cause disease. GWAS consistently identified common genetic variants of *SNCA* to be associated with increased risk of PD [64, 66]. Missense mutations are located in the N-terminal region of the protein which normally folds into a helical conformation to bind to neuronal synaptic membrane. These mutations result in a variety of structural effects that may promote oligomer formation and loss of membrane binding [67]. A large body of evidence supports the concept that formation of α-synuclein oligomers and fibrils that are ineffectively cleared by the lysosomal system plays a key role in PD pathogenesis. This has led to exploration of potential therapeutic approaches by strategies including reduction of α-synuclein production, inhibition of α-synuclein aggregation, enhancement of autophagy, reduction of extracellular α-synuclein and inhibition of cellular uptake of α-synuclein [68].

Mutations in *leucine-rich repeat kinase 2* (*LRRK2*) were first identified in a Japanese family [69] and subsequently independently in several families in different countries; these mutations represent one of the most common genetic causes of PD [82]. *LRRK2* mutations are relatively common and has been estimated to be present in 40% of familial cases and 10% of sporadic cases worldwide [65]. The most common *LRRK2* mutation, p.G2019S, has incomplete penetrance and is found in 4% of familial and 1% of sporadic PD cases [83]. This same mutation, along with other variants in *LRRK2*, has also been identified as a susceptibility locus in sporadic PD [84]. The LRRK2 protein contains domains that include a GTPase and kinase function, along with many other protein/protein interaction motifs [85]. It appears to be important in several cellular pathways, such as vesicle trafficking, endosomal maturation, membrane dynamics, autophagy and mitochondrial function [85]. Animal models with mutant LRRK2 exhibit synaptic deficits such as reduced vesicle endocytosis [86], and altered dopamine release, uptake and signaling [87, 88]. LRRK2 is also a binding partner of the late endosomal marker Rab7 and the lysosomal marker LAMP2A, suggesting a role of LRRK2 in the endo-lysosomal pathway [85]. A mutant LRRK2 cell model showed reduced lysosomal function and reduced clearance of chaperone-mediated autophagy substrates, including α-synuclein [89]. LRRK2 is involved in mitochondrial membrane maintenance by increasing expression and phosphorylation/activation of Drp1 which promotes mitochondrial fragmentation and fission [90, 91]. The pathogenicity of *LRRK2* mutations is likely mediated by an increase in kinase function, as it has been shown that these variants lead to increased kinase activity [70–72]. Furthermore, there is evidence of increased LRRK2 kinase activity in the brains of idiopathic PD patients [92], suggesting that LRRK2 kinase inhibitor may be a viable therapeutic option in the treatment of PD. To this end, progress has been made in the development of assays to measure in vivo LRRK2 kinase activity and to evaluate the therapeutic potential of LRRK2 inhibitors in PD [73, 74].

The discovery of *glucocerebrosidase* (*GBA*) mutations in PD originates from the observation of increased incidence of parkinsonism in patients and obligate mutation carriers in families of Gaucher disease (GD), an autosomal recessive lysosomal storage disease caused by homozygous and compound heterozygous *GBA* mutations which lead to reduced glucocerebrosidase (GCase) enzymatic activity [75, 76]. A large multicenter study subsequently confirmed the association between *GBA* mutations and PD and found that *GBA* mutations are significantly more prevalent in PD compared with controls, with an odds ratio of 5.43 [93]. Approximately 5–10% of PD patients have *GBA* mutations, making this gene the most important genetic predisposing risk factor for PD. The prevalence of *GBA* mutations vary widely among different ethnicities, with the highest frequency of 10–31% found in Ashkenazi Jewish PD patients [77]. In Asian populations, the frequency of GBA mutations is 3.72% in Taiwanese Chinese patients [78], 8.7% in mainland Chinese patients [79], and 9.4% in Japanese PD patients [80]. Furthermore, there is heterogeneity in the variants identified in *GBA* among different populations. For example, the N370S mutation, which is the most commonly found one in Ashkenazi Jews, is rare in Asians, whereas the L444P variant is the most common in Chinese PD patients. Lewy bodies identical to those observed in idiopathic sporadic forms of PD have been found in patients with neuronopathic GD, suggesting that α-synuclein aggregation is involved in the neurotoxicity of GD [94]. Furthermore, studies of idiopathic sporadic PD brain have shown that GCase expression and activity are decreased in brain regions that accumulate α-synuclein, supporting the relevance of reduced GCase activity in the development of α-synucleinopathy even in patients expressing wild-type GCase [95, 96]. The mechanisms by which *GBA* mutations result in PD are not well understood, and both loss-of-function and toxic gain-of-function hypotheses have been proposed, although neither models are perfect. The current leading hypothesis posits that reduced GCase activity leads to accumulation of its substrates, resulting in toxic build-up of insoluble α-synculein and compromise of lysosomal function. α-Synuclein then impairs trafficking of GCase from Golgi and endoplasmic reticulum (ER) to the lysosomes, exacerbating the build-up of substrates, forming a self-propagating bi-directional loop [81]. The fact that build-up of α-synuclein has been found to result in GCase deficiency, in the absence of mutant enzyme, holds significance for the wider PD population. Efforts have been underway to explore modulation of GCase activity as a therapeutic strategy for GBA-PD and conceivably for sporadic PD as well. Strategies include brain-penetrating small molecule chaperones to increase lysosomal GCase activity and reduction of GCase substrate buildup by glucosylceramide synthase inhibitors [68].

Another gene of interest in PD is *MAPT*, which encodes for microtubule-associated protein tau. Mutations in *MAPT* were first associated with frontotemporal dementia and parkinsonism linked to chromosome 17 [97]. Subsequently, GWASs and their meta-analyses have also identified *MAPT* as a risk factor for sporadic PD [84]. *MAPT* lies in a long region of linkage disequilibrium with two variants: the directly oriented haplotype H1, and the H2 haplotype containing an inverted 900-kb chromosomal sequence which is present in 20% of Caucasians but is almost never found in Asians [98, 99]. The haplotype H1 is associated with increased risk for PD and homozygous H1/H1 genotype is a biomarker of dementia in PD [100]. Of note, neurofibrillary tangles consisting of phosphorylated tau can be found besides Lewy bodies in PD patients with more pronounced cognitive decline [100]. It has been shown that α-synuclein influences tau phosphorylation and fibrillization [101], suggesting an interplay between these two genes which can influence risk of PD. Indeed, carriers of combined *MAPT* H1/H1 and *SNCA* rs356219 G/G genotype have been shown to have double the risk of PD [102]. These discoveries of causal and risk genes associated with PD mean that it is now possible to study larger cellular networks in which these genes may interact to influence phenotype. For example, pathway analysis of the nominated candidate genes identified in GWASs has led to the discovery of several major biological pathways implicated in PD: autophagy, endocytosis, mitochondrial biology, immune response and lysosomal function [83]. Identification of these pathways will in turn inform future strategies in targeting these pathways in the treatment of PD.

Undoubtedly, the advent of massive parallel sequencing and new genetic analysis techniques has uncovered a much higher genetic contribution to the etiology of PD than previously thought since the first discovery of *SNCA* 20 years ago. However, genetics alone is unlikely to be a cause in the majority of PD patients. *SNCA* missense and multiplication mutations are rare [59], and more commonly seen mutations in *LRRK2* and *GBA* have incomplete, age-dependent, penetrance with some carriers living to old age without symptoms of PD [83]. Thus, the relative contribution of pathogenic Mendelian genes to PD is limited. Common polymorphisms identified in GWAS individually confer only modest risk. Collectively, the heritability of PD has been estimated to be about 30% [103]. Clearly, additional factors such as aging and the environment are involved in PD pathogenesis.

### Interplay of aging, genetics and environment: what animal models show

Aging is recognized to be the primary risk factor for PD with incidence rising exponentially with advancing age. Nevertheless, most elderly over 85 years do not have PD. Familial PD due to monogenic causes account for about 5% of all PD but the majority of known mutations have incomplete and variable penetrance. It is hypothesized that genetic risk factors may render the individual more sensitive to the pathologic influence of other factors. For example, the cumulative risk of PD in carriers of *LRRK2* p.G2019S, the most common *LRRK2* mutation in PD, rises exponentially from the age of 50 onwards [104], suggesting that aging interacts with *LRRK2* mutations to initiate disease. This observation is corroborated in a *LRRK2* G2019S knock-in (KI) mouse model which showed dysfunctions in plasma membrane and vesicular DA transporters, and accumulation of serine129-phosphorylated α-synuclein, the predominant form of α-synuclein in Lewy bodies, in 12-month-old KI mice compared to age-matched wild-type (WT) mice. These changes were not present in young 3-month-old KI mice, suggesting a progressive response relying on the interaction of the mutation and aging [105]. Similarly, another *LRRK2* KI mouse model, *LRRK2* R1441G, showed a progressive, age-dependent accumulation of oligomeric α-synuclein in the striatum and cortex compared with age-matched WT mice; this higher rate of oligomeric α-synuclein accumulation in KI than WT mice was first apparent at 15 months and became significant at 18 months of age [89].

Environmental toxins as a cause of parkinsonism have been recognized since the discovery of MPTP-induced PD. However, most PD patients do not have significant history of acute exposure to these toxins. Furthermore, it has been observed that a single heterozygous mutation in the recessive *PINK1* and *Parkin* genes could lead to PD in some patients [106], raising the possibility that these heterozygous mutations increase susceptibility to environmental toxins that collectively become sufficient to cause PD. The gene-environment interaction has been documented in a number of experimental models. For example, primary neuronal cultures of *Parkin* knockout mice were much more sensitive to the toxicity of rotenone, a potent mitochondrial complex I inhibitor found in pesticides, compared with wild-type mice [107]. In young *LRRK2* R1441G KI mice under normal conditions, there are no differences in striatal synaptosomal DA uptake, locomotor activity, and amount and morphology of nigrostriatal DA neurons and neurites compared with controls. However, after exposure to a single intraperitoneal dose of reserpine, a reversible inhibitor of vesicular monoamine transporter-2 (VMAT2), these young mutant mice exhibited lower DA uptake with impaired locomotor activity, as well as a slower recovery from the effects of reserpine, compared with age-matched young WT mice [88]. Similarly, primary cortical neurons, mesencephalic DA neurons and striatal synaptosomes from young KI mice were found to be more susceptible to rotenone toxicity, resulting in increased cell death, ATP depletion and reduced synaptosomal DA uptake [108]. Because many genetic models of PD exhibit impaired mitochondrial function, and environmental toxins have also been shown to affect mitochondria, mitochondrial dysfunction has been proposed as the common pathogenic pathway of both PD-linked genetic mutations and environmental toxins, along with other non-mutually exclusive mechanisms which include oxidative stress, neuroinflammation and impaired autophagy [60].

A study examined the combined effects of genetic mutation, aging and chronic sub-lethal environmental toxin exposure. Thirty-month-old *LRRK2* R1441G KI mice treated with repeated low doses of oral rotenone twice a week for 50 weeks had greater reduction in locomotor activity compared with similarly treated wild-type mice. In addition, these rotenone-treated KI mice exhibited a significant reduction in striatal mitochondrial complex I levels, which was not observed in similarly treated wild-type mice [108]. This unique model captures the interplay among aging, genetic susceptibility, and chronic environmental toxin exposure which are important in the pathogenesis of PD, a typically late-onset, slowly progressive neurodegenerative disorder.

### Conclusions and future directions

**Fig. 2:**
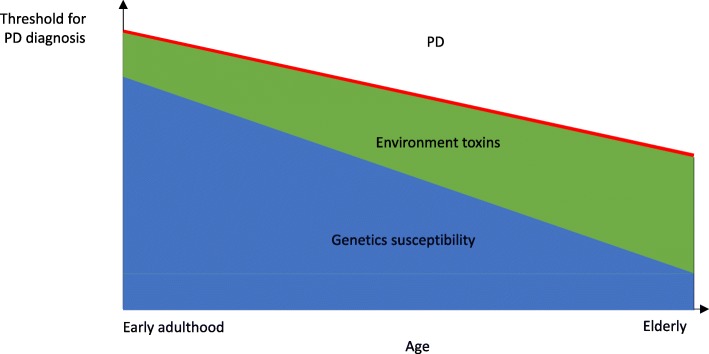
Schematic diagram of the interplay among aging, genetic susceptibility and environmental toxins in the pathogenesis of PD. The red line represents the threshold of DA neuronal stress over which PD will manifest. With increasing age, impairment in cellular pathways such as oxidative stress, mitochondrial dysfunction increases the susceptibility of DA neurons to degenerate and thus lower the threshold for developing PD. During younger age, genetic susceptibility likely plays a more major role in causing PD, whereas cumulative exposure to environmental toxins likely has more significant contribution to PD development in old age

After Dr. James Parkinson’s first description of PD in 1817, the clinical features and anatomopathological correlates became well recognized in late nineteenth century and the first symptomatic oral treatment, levodopa, became available in 1960’s. However, the etiology of PD baffled physicians and scientists until 2 breakthrough discoveries: MPTP-induced parkinsonism in 1983 and the discovery in 1997 of the first genetic mutation in PD: *SNCA*. The recognition of α-synuclein being the major constituent of LB led to the hypothesis that α-synuclein spreads through different regions of the brain through six pathological stages of the disease [58]. The rapid advancement in the field of PD genetics has not only shed light on cellular pathways that are involved in PD and provided potential targets for intervention, but also facilitated the generation of multiple genetic animal and cell models of PD. However, limitations remain as there are currently no animal models that replicate completely the α-synucleinopathy seen in human PD. Transgenic α-synuclein models that overexpress the mutant or WT protein develop α-synuclein aggregates but commonly do not have the nigral neurodegeneration seen in human PD. Toxin-based animal models are based on acute exposure to high doses of neurotoxins which do not reflect the typically late-onset, slowly progressive degenerative process in human PD. Aging is necessary to induce expression of the DA neurodegenerative phenotype in human iPSCs and is clearly important in PD pathogenesis, but is difficult to be incorporated into animal models. Notwithstanding these limitations, multiple genetic-based animal models have shown that genetic mutations alone are rarely sufficient to replicate the pathologies seen in PD and that additional challenges with aging and/or environmental toxins are necessary. It is thus clear that PD is a multifactorial, heterogeneous group of disorders, with genetics, aging and environmental exposure interacting in different ways in different patients. The risk of PD in an individual will depend on genetic susceptibility in terms of the presence and burden of pathogenic variants and common risk variants, which will influence the susceptibility to environmental factors and the effects of natural aging [Fig. 2].

Despite the understanding of the necessary components of PD etiology, much remains unclear. Some common and converging pathways that are affected by genes strongly associated with PD, aging and environmental toxins include mitochondrial dysfunction, oxidative stress, neuroinflammation and impaired autophagy with accumulation of misfolded proteins. It remains to be seen whether these pathways can be effectively targeted to treat PD. Dopaminergic neuronal loss starts 20–30 years before the first motor symptoms appear, by which time there is already 50% striatal dopamine reduction [109]. Therefore, there is a long prodromal or pre-motor period during which non-motor symptoms such as hyposmia and REM sleep behavior disorder already start to emerge [48]. This long prodromal period is compatible with the view that the progression of PD is likely to require multiple hits and may present a window of opportunity when the pathogenic processes may be slowed or stopped. Understanding of the events and pathways that drive the initiation and progression of PD will shed light on possible targets for disease modification.

## Data Availability

Not applicable.

## Abbreviations

ER: Endoplasmic reticulum
*GBA*: *glucocerebrosidase*
GCase: Glucerebrosidase
GCTA: Genome-wide complex trait analysis
GD: Gaucher disease
GWAS: Genome-wide association study
iPSC: induced pluripotent stem cells
KI: Knock-in
LB: Lewy bodies
*LRRK2*: *leucine-rich repeat kinase 2*
MPTP: 1-methyl-4-phenyl-1,2,3,6-tetrahydropyridine
PD: Parkinson’s disease
RBD: REM sleep behavior disorder
SN: Substantia nigra
TH: Tyrosine hydroxylase
VMAT2: Vesicular monoamione transporter-2
WT: Wild-type
